# The Barriers and Facilitators to Decentralised Nurse-Led Mental Health Service Delivery in Sierra Leone

**DOI:** 10.1007/s10597-023-01087-0

**Published:** 2023-01-24

**Authors:** Helen Hopwood, Dawn Harris, Stephen Sevalie, Gloria Iyawa, Julie Langan Martin

**Affiliations:** 1 King’s Centre for Global Health and Health Partnerships, School of Population and Environmental Sciences, King’s College London, London, UK; 2 Regimental Military Hospital, Freetown, Sierra Leone; 3 Kings Sierra Leone Partnership, Freetown, Sierra Leone; 4 Sustainable Health Systems, Freetown, Sierra Leone; 5 University of Salford, Salford, UK; 6 School of Health and Wellbeing, University of Glasgow, Glasgow, UK

**Keywords:** LMIC’s, Traditional healing, Monitoring and evaluation, Motivation

## Abstract

In 2009, 98.0% of people with mental illness in Sierra Leone were not receiving treatment, partly due to the absence of public psychiatric facilities outside the capital. In response to this situation, the Ministry of Health and Sanitation rolled out nurse-led mental health units (MHUs) to every district. This study evaluates the barriers and facilitators to mental health service delivery in decentralised MHUs in Sierra Leone using key informant interviews and focus group discussions with 13 purposefully sampled clinical staff and senior management personnel. The interviews were audio-recorded, translated from Krio if necessary, transcribed, and analysed using manifest content analysis. The findings suggest that factors affecting nurse-led mental health service delivery include small workforce and high workload, culture and beliefs, risks, lack of safety measures and required resources, outdated policies, poor salaries, lack of funds for medication, distance, power, influence, and stigma. Factors that could facilitate nurse-led mental health services include: increasing motivation, increasing the workforce, knowledge sharing, mentorship, availability of medication, passion and modern psychiatry. The findings contribute towards understanding the challenges and opportunities faced by the recently established nurse-led decentralised mental health services across Sierra Leone, in order to address the large mental health treatment gap. We hope the findings will inform further policy and planning to improve the quality of decentralised mental healthcare.

## Background

Mental illness constitutes a huge global burden of disease (Rehm & Shield, 2019), but in low-income and middle-income countries (LMICs) there is a large treatment gap, ranging from 76·3 to 85·4% (Demyttenaere et al., 2004). The gap is generally attributed to challenges related to human resources, stigma, political factors, technology and infrastructure (Shrivastava et al., 2012).

Sierra Leone is in west Africa. It has a population of 7.5 million (World Bank, 2020b). The country experienced civil war between 1991 and 2002 which destroyed infrastructure including healthcare systems. With a gross national income per capita of $496.7 in 2017, it is classed as a low-income country (World Bank, 2020a). The population is young, with 41% aged under 15 (World Bank, 2020b). Sierra Leone was among the countries most affected by Ebola Virus with 8,706 people infected and 3,590 deaths, including many of the healthcare personnel (World Health Organisation, 2021).

In 2009, in Sierra Leone, the treatment gap for mental illness was 98·0% (Alemu et al., 2012), partly a result of there being only one Ministry of Health and Sanitation (MOHS) treatment facility, the Sierra Leone Psychiatric Teaching Hospital (SLPTH, Formally known as Kissy Mental Hospital or Kissy Lunatic Asylum) in Freetown. Sierra Leone is likely to have a high prevalence of mental illness; a 2002 Ministry of Health and Sanitation (MOHS) survey conducted at the end of the civil war indicated that depression (4%) and severe substance abuse (4%) were the most common mental health difficulties, followed by psychosis (2%), and epilepsy (1%) (Alemu et al., 2012).

In order to increase treatment coverage, in 2012 the MOHS collaborated with partner organisations to train 21 nurses in a 12–18 month mental health program (Enabling Access to Mental Health in Sierra Leone, 2011; Ministry of Health & Sanitation, 2010). In 2015, these nurses went on to establish mental health units (MHU) in all 14 districts of Sierra Leone. The nurses provide treatment guided by the World Health Organisation’s (WHO) mental health Gap Action Plan (mhGAP) (World Health Organization, 2010). The mhGAP proposes that access to mental health treatment can be improved by health system restructuring and task-sharing with nurses and non-specialists. Some supervision is provided by NGOs and local psychiatrists, although there are challenges of access and sustainability, especially given the cost and time involved in travelling between districts. The nurses work with the doctors in their hospital to have prescriptions written.

Internationally, the mhGAP model has been adopted in over 90 countries but has had limited evaluation in many places. Research into task-sharing with non-specialists in the sub-Saharan region is scarce, outside of primary care (Keynejad et al., 2018). Suggested barriers towards scaling up non-hospital psychiatric care include the low prioritisation of mental health in the public health agenda, historical reliance on inpatient care and resistance to community treatment, large workloads for staff with limited specialist support and poor medication supplies (Saraceno et al., 2007). Additional factors relate to the extent to which organisations are open to implementation, such as the knowledge and attitudes of staff, needs of the patients and apparent benefits from treatment (Esponda et al., 2020).

Sierra Leone’s MHUs are accessible to both adults and children. Initial evaluation of these units has demonstrated that primarily patients with epilepsy or psychosis access the service (Hopwood et al., 2021). Despite an increase in demand, these MHUs have so far had a relatively small effect on the treatment gap. Furthermore, there is wide variation in the number of referrals into different MHUs, which appears un-related to district population.

Identified challenges in Sierra Leone include the MOHS not having specific budget line for mental health outside SLPTH, which is noted to have its own funding difficulties (Harris et al., 2019). Additionally, there are also sizeable problems with medication supply throughout the country. Workforce shortages are also a significant challenge in all sectors of the healthcare system (Sierra Leone Ministry of Health & Sanitation, 2016). The majority of the MHUs are staffed by a single nurse working alone, responsible for all clinical and service management. Multi-disciplinary support is limited. In SLPTH there are three psychiatrists. One further psychiatrist is posted in a military hospital, also in Freetown. There are no public psychotherapy services, no occupational therapists or speech and language therapists. There is one mental health social worker, based in Connaught Hospital in Freetown. Within primary healthcare, mental health knowledge and awareness is low, although recently some primary health unit staff (Community Health Officers, CHO) have accessed five-day training programmes in mhGAP (Bah et al., 2018). The impact of these challenges on the effective delivery of mental health services is unknown.

Given that decentralisation of services is a key global priority to address the burden of mental health disorders (World Health Organization, 2003), evidence is needed on the specific barriers and facilitators in Sierra Leone to delivering care within these new decentralised services, both in the day-to-day running of the units at the district level and from the managers making planning decisions at higher level. This evidence will guide future directions in how to use the limited resources available to build capacity in the system and further reduce the treatment gap through policy, planning and implementation. The aim of this study was to investigate the barriers and facilitators to mental health service provision at service and management levels within Sierra Leone’s district nurse-led MHUs.

## Methods

### Setting

The 15 MHUs are based in each district of Sierra Leone in 12 district general hospitals and three in Freetown: one general referral hospital, one children’s hospital and one military hospital. At the time of undertaking this study, there were 18 trained mental health nurses staffing these units. The units assess and manage patients with signs of any mental health, substance misuse or neurological conditions, for example depression, psychosis, epilepsy, or intellectual disability. The management of epilepsy is supported by a neurologist who has an epilepsy clinic in Freetown. In the first two years following their implementation, in 2015 and 2016, these MHUs saw a total of 2400 patients (Hopwood et al., 2021 Apr). There was an average of four new referrals a month in each unit from any source, including self-referrals, community and hospitals.

### Participants

Sampling was purposive, with the aim of recruiting participants with a range of job roles and experience, within front line patient facing roles and MOHS roles with an overview of the service delivery. The total number of participants was 13: four women (who were all mental health nurses) and nine men. A focus group discussion followed by key informant interviews were conducted with eight mental health nurses. The aim of using the focus group was to identify themes that could be further investigated in more depth during the individual interviews, where it was observed that participants were more vocal (Mishler, 1986). Three participants were recruited for key informant interviews from senior management positions within the MOHS: the Chief Pharmacist, Monitoring and Evaluation (M&E) Officer and Scientific Officer. One psychiatrist at the Sierra Leone Psychiatric Hospital and the Country Director for Traditional Medicine were also interviewed. The focus groups were not repeated for these participants because there were practical restraints to bringing all participants together.

### Interviews and Focus Group

One focus group and 13 interviews were carried out between January and March 2017. The focus group was facilitated by research assistants, with oversight from a member of the research team who was a psychiatrist based within the military hospital. The interviews and focus group were held in Freetown, during training and academic events, therefore the participants were already released from their clinical duties and available in Freetown. Participants received no financial compensation for their participation. Informed consent was obtained using an informed consent document. Some participants may be identifiable from their answers, due to their position, which was made clear during the consent process. The interviews and focus group used a semi-structured interview guide, with questions assessing current service delivery in mental health, and how the workforce, physical environments, organisations, tools and tasks are barriers and facilitators to mental healthcare delivery in decentralised MHU. Interviews lasted about 30 min and the focus group lasted 60 min. Interviews were conducted in a mixture of Krio and English and audio-recorded.

### Data Analysis

Interview and focus group recordings were translated (if necessary) and transcribed. Transcripts were imported into NVivo software for coding and analysis (Braun & Clarke, 2006). Three investigators (GI, HH and DH) conducted content analysis. The investigators read the data several times, became familiar with the discussions and deduced manifest meanings from the text. Units of meaning (verbatim quotes) were coded and codes that related to the same item were merged into categories. Categories that belonged to each other were given a heading, revealing the core themes. Then all three authors discussed the categories and themes until agreement was reached on the naming. The process was repeated until data saturation was reached and all investigators identified no new codes. Codes were then grouped into potential barriers and facilitators and inspected for overlap to ensure they were defined and distinct from each other. Finally, the investigators selected key quotes for each barrier and facilitator.

## Results

Several key themes were identified relating to the barriers and facilitators to decentralised nurse-led mental health service delivery in Sierra Leone. These are summarised in Table 1 and 2 respectively.

**Table 1:** Themes relating to barriers

Theme	Subtheme	Example quote
Challenging working conditions	Small workforce	“…we encouraging and appealing to the ministry of health and sanitation to continue training more mental health nurses in the country. Because we are about eighteen or nineteen remaining: two died” (KII Participant 7)
	High workload	“Our caseload in some districts is very high” (KII Participant 4)
	Large catchment areas/travel distances	“The district that I cover is a big district” (KII Participant 4)
	Lack of safety measures	“…we have high risk patients around us … But I don’t have that kind of security, which I think can help me when I need help” (KII Participant 5)
Culture, beliefs and stigma	Lack of mental health literacy	“People still believe mental illness is caused by demons, by witchcraft and so on and so forth” (KII Participant 2)
	Stigma	“When somebody gets some problem especially in mental health first of all he is isolated in some social activities. Sometimes [people] ignore him in getting a marriage to Mr A and B” (KII Participant 13)
Lack of resources	Outdated policies	“We need a very robust service policy” (KII Participant 2)
	Poor salaries	“…they don’t want to go there because there is no money and there is no motivation” (KII Participant 7)
	Lack of funds for medication	“…yet they will not comply or give money to buy drugs” (KII Participant 6)
	Power and influence	“If I want any utensils, drugs or whatever, the chain of command is for the person responsible to write through the official channel through the psychiatrist in charge and then it goes to the responsible person in the Ministry, the HQ” (KII Participant 2)
	Lack of integrated care/monitoring and evaluation systems	“It has been a very serious challenge getting everybody on board to understand that there is benefit in having an integrated M&E system” (KII Participant 11)
	Lack of support for traditional medicine	“We need total cooperation for our traditional mental health doctors” (KII Participant 13)

**Table 2:** Themes relating to facilitators

Theme	Subtheme	Example quote
Motivation	n/a	“Like I am opportune to have Kings with me and they are helping me, and the hospital that I am working in, they are also helping me, they are trying their bit. We have JSI (John Snow Inc.)” (KII Participant 3)
Knowledge sharing	n/a	“Whatever workshop I attend, I will make sure that I find time to teach them or share the knowledge with them” (KII Participant 5)
Passion	n/a	“Just that I have passion for the job and the patients that I am treating; if not I should have given up from this mental health work long since” (FGD Participant 6)
Building capacity	Increasing the workforce	“If I were the one implementing this service, I should have brought a lot of people on board, that is the first step” (KII Participant 6)
	Modern psychiatry	“Expose them to modern psychiatry and increase funding” (KII Participant 12)

Participants 1–8 are mental health nurses and participants 9–13 are the other key informants.

### Barriers

#### Challenging Working Conditions

Challenging working conditions were highlighted as a key barrier to successful decentralised nurse-led mental health service delivery. In particular issues with the small workforce, high workload, covering large catchment areas and travelling distances, working in isolation and lack of safety measures were highlighted as particular issues.

#### Small Workforce and High Workload

Out of the 21 mental health nurses originally trained in country, two have passed away, leaving 19 mental health nurses available (Harris et al., 2019). Death was listed as one of the key factors affecting capacity building and as such, nurses explained that it was necessary to train more mental health nurses. A decline in the size of the workforce could lead to high caseloads. Furthermore, other nurses also indicated that some of the nurses left in the workforce are almost at retirement age calling for a need to train more mental health nurses.

Am also appealing that some of us are on the pathway now actually to retirement, so we are encouraging and appealing to the Ministry of Health and Sanitation to continue training more mental health nurses in the country. Because we are about eighteen or nineteen remaining: two died. (KII Participant 7)

Nurses in certain districts reported high caseload as a challenge which often affects their work activities. Nurses also believe that they would be taken more seriously if they were greater in number. Furthermore, nurses reported poor motivation. This was often attributed to a lack of recognition received for their work from the MOHS. The participants also highlighted that as a result of not being supervised, they find it difficult to take work seriously. Some of the participants explained:

Our caseload in some districts is very high in a way that for us to even manage ourselves is difficult, and more also the caseload and work is plenty for us. This has made us psychologically out of tract, because of caseload, no motivation, no encouragement and no one is checking us to know what is happening … To let a single mental nurse cover a whole district is not easy thing, and no one cares about you, even if you are sick, they don’t care … No one is coming out of the Ministry to check on us. When we hear supervisors are coming, we too will take our work seriously. We take our supervisors to our success cases… (KII Participant 4)

Also lack of expanding the current facility beyond Freetown; we only have one mental home in Freetown and the service needs to be extended to the provinces at least at district levels… (KII Participant 10)

#### Large Catchment Areas/Travel Distances

Sierra Leone is split into 14 districts. These districts can cover large geographical areas and varying terrain. Outside of Freetown, each district has only one MOHS operated mental health unit to service the entire population. Some patients are located in distant locations and are unable to reach the district hospitals, leaving them without access to mental healthcare. Another problem encountered is the lack of ambulance services, which adds to the difficulty in transporting patients. One nurse explained:

The district that I cover is a big district. Some of the problems that we encounter in our everyday work are: there are some areas where the patients don’t have access to those places and some of them are very violent. So, it can be very difficult to bring them. (KII Participant 4)

#### Lack of Safety Measures

It was mentioned that nurses are sometimes left in a vulnerable position when treating acutely unwell patients in isolation. However, there is a lack of safety measures and risk assessments in place. It was mentioned that there is a lack of security personnel, essential medications as well as a secure environment to support high risk patients who can harm themselves and others. Sometimes, the nurses have been promised medical supplies but not received these.

In most cases the patients that we normally become friend[s] with, like trying talk with them for them not be alone or left out, usually sometimes return to their problem, in which they will at times give us a slap or hit us with something. This is what I fear because we have no risk allowance policy from the Ministry of Health and Sanitation, they don’t value us as mental health nurses … So I believe that actually it is God’s protection that is over us, and with the passion and love we have for the job maybe it is through God seeing that that protects us; but if anything negative does happen to us out there that is risky and our family will face the consequences. (KII Participant 4)

Accessing the community at large can be challenging, because me being a woman and I have a motorbike, I am unable to ride a far distance. But the chiefdoms around the district in my township, I used to go there and talk with the community people and whenever, like I’m saying, if we have high risk patients around us … But I don’t have that kind of security, which I think can help me when I need help. So, if I have that kind of case I will not allow him in my office until I have strong and able men around before I do my work. Sometimes, although it is not right to treat them by force, but we will make sure that people are around that will help us hold onto them, then we carry out our work. (KII Participant 5)

### Culture, Beliefs and Stigma

Another prominent theme highlighted as a barrier was that related to culture, beliefs and stigma. In particular poor mental health literacy and stigmatising views were identified as key barriers.

#### Lack of Mental Health Literacy

Some of the participants explained that there is a huge gap in terms of mental health literacy levels amongst the general population. Many people hold the belief that mental health disorders are caused by witchcraft or demons. As a result, people usually opt to first seek help from unregulated faith based and traditional healers (Jones et al., 2009). This often leads to a delay in presentation to government mental health services, extending the duration of illness and increasing the workload for the nurses. However, participants explained that they come from the same culture and understand and respect the community beliefs. Some of the participants explained:

People still believe mental illness is caused by demons, by witchcraft and so on and so forth, so the first point of call is to the traditional healer after they have been deprive[d], they have been physically abused sometimes. At the end of the day the relatives deem it fit to bring them to the hospital or by the time they come they are … in a chronic state … so that you have to be very professional to bring them out of that problem. (KII Participant 6)

Of course, it is always both cultural and normal. People, normally out of necessary sympathy, people attach a lot of negative things with people with mental health. Some see it as any normal illness. Although there are some people that do show some sympathy and normally at domestic level but in the society, they don’t normally contribute much towards the care of mentally affected [people]. We need to focus as a country, even the Ministry needs to improve the advocacy so that mental health could be taken seriously as a health problem. (KII Participant 10)

They have those awareness and then we are respecting every belief and we are going strictly by those norms. We are not frowning at any one opinion and we normally do come in to talk in order to have a common ground as to how we could help the people. (KII Participant 4)

#### Stigma

Mental health service delivery is often stigmatised. As a result, mental health services are often given low priority compared to other health services. This is often a source of discouragement for these nurses. Patients are also stigmatised even after they have been treated. Some of the participants added:

We all belong to tribes. When somebody gets some problem especially in mental health first of all he is isolated in some social activities. Sometimes [people] ignore him in getting a marriage to Mr A and B and even taking part in political arena, some even are not encouraging him to take part in other areas so what we do we start to talk to them. We held a series of workshops that no Mr B has gotten this problem but now we thank God he has got better. He has to be integrated and please don’t stigma him because he has a big problem. (KII Participant 13)

They have activities that they are priority, so imagine when you wanted to do an activity and they will side-line saying let’s wait (mental health) they have important activities to deal with. (KII Participant 5)

### Lack of Resources

Finally, lack of resources was identified as a key barrier to successful implementation of the nurse-led mental health services. Lack of resources included issues with outdated policies, poor salaries and lack of access to medication.

#### Outdated Policies

Sierra Leone continues to use the Lunacy Act of 1902 for its mental health legislation. This legal framework is outdated and does not address the current challenges faced in mental healthcare. In 2012, the first ever national Mental Health Policy was launched in Sierra Leone (Bah et al., 2018). At the time of the participant interviews, this policy was being updated. Some of the participants raised concerns regarding creating new policies and strategies as well as ensuring these policies are converted into legislation and enacted. It was also recommended that these policies should incorporate the current needs of mental healthcare in Sierra Leone such as infrastructure and safety.

We need a very robust service policy, am sorry because with policy, if this policy are enacted then mental health service will be high up, there will be at par with other services … but we need a very good policy in terms of emulation, in terms of motivation, in terms of conditions and development, in terms of encourage other staffs from other disciplines to come and work in the hospital. (KII Participant 2)

But sometimes it comes to implementation, you know the Ministry or staff would have sat down and come up with very good strategy but yet we need funding to promote or to implement. And if not, then what happens, you know things haven’t worked, you know it is just support, you know in implementation because really it is the key for most of the things to be done. (KII Participant 9)

#### Poor Salaries

It was identified that mental health nurses are not well paid and that this additionally contributes to their poor motivation. The 19 nurses completed either the diploma or certificate in mental health nursing in 2013, but many of them have not received any financial increment in their salary to reflect their additional qualifications. One or two are not being paid due to reasons related to problems within the Government and central HR processes. Nurses must often fund their phone credit, printing and petrol themselves. Subsequently, other healthcare professionals are unwilling to join the mental health workforce because of the poor salaries. One of the participants stated:

I thank you so much for that important question. If I was in place to promote mental health in this country, I think foremost of all this is a no go area for our other colleagues in the medical profession because they don’t want to go there because there is no money and there is no motivation. (KII Participant 7)

Since they posted here in the district I have not been on salary, it is two years and six months now that I have been volunteering as a mental health nurse in district with no salary. The job is not really easy, more the kind of district that I found myself talking about - the road network, which so bad, and even the sacrifice we are making… (KII Participant 6)

#### Lack of Funds for Medication

Mental health medications are not included in the national free healthcare initiatives by the MOHS. Patients often complain that there is no money to purchase medications. This is a significant challenge to providing adequate care. Mental health medications are not free, and the cheaper medications may be less tolerated than costlier alternatives. It was also highlighted that there are lots of fake medicines in the pharmacies. Occasionally, nurses will use their own money to buy medications for patients, which is a constraint for these nurses. Some of the participants stated:

These relatives normally complain that there is no money, and they know there is no free treatment for mental health, yet they will not comply or give money to buy drugs, so sometimes it is we the mental health nurses that squeeze out the little money we have to buy drugs for them (KII Participant 6)

…government is trying to procure but we have not still got from them yet…. We have pleaded for assistance in order for us to have medications. (KII Participant 8)

It has to deal with the appropriate manpower, equiptly trained and also in number available, in the continuous access to the appropriate drugs, medical supplies, at all times and not only at Kissy but also the services need to be extended at district level and even up to PHU [Primary Healthcare Unit] level. (KII Participant 10)

#### Power and Influence

One of the participants explained that power and influence may have an impact on service delivery since approval for certain resources including medications often takes time because it must go through different phases of approval at management level. Participants also believe that their views are discounted because they work as mental health workers, irrespective of qualification.

Participant 10’s main contributions (apart from stigma) are to say what should be extended to the districts, and that he doesn’t know whether that has happened.

Participant 9 mostly talks about not knowing much about mental health (e.g.) “I only heard that services are really poor but a lot of work has been done recently by the … organization like WHO, Kings and other organizations to improve mental health services, even training nurses to deliver mental health services that I think is in the right direction, as I have been in previous meeting with various stakeholders including this traditional healers trying to promote this whole thing on mental health. So, I think it in the right direction…”.

Participant 10 was impressed by hearing some information about mental health during a talk delivered one of the nurses.

If I want any utensils, drugs or whatever, the chain of command is for the person responsible to write through the official channel through the psychiatrist in charge and then it goes to the responsible person in the Ministry, the HQ and then from there we can then be supplied, sometimes we are not, sometimes it takes quite some time, sometimes the availability is a problem to the healthcare delivery service. (KII Participant 2)

We have rendered all our efforts and services, but we are not seeing any better facilities for us. (FGD Participant 7)

What I’m saying there is no political will in terms of mental health. They can only call you when there is a crisis in the community, they will start calling (saying) this patient has stabbed so so person what are you guys doing there… (KII Participant 12)

#### Lack of Integrated Care/Monitoring and Evaluation Systems

Participants in management positions indicated that the lack of understanding of mental illness and the healthcare service, as well as the government not taking mental health seriously but paying more attention to wealthier programs constitutes another challenge. It was also indicated that there is a lack of monitoring and evaluation systems in place. One manager explained:

It has been a very serious challenge getting everybody on board to understand that there is benefit in having an integrated M&E system …, so it was difficult. Some programs are stronger than the others in terms of finance, human resources, so they tend to dominate. (KII Participant 11)

I have to do monitoring, moving from one point to the other by using my motorbike. Gas is provided by me or the Executive or the President of the Traditional Healers Union … and even the maintenance is done by me. I need a vehicle at least to make my movement very fast to get to traditional healers that deal with mental health so we can get them totally incorporated into the system. (KII Participant 13)

So it is like when it does really reflect, we are appealing to the Ministry and country as a whole for us to review this particular M&E, from that we will capture all our cases that we see and it really needs to be reflected in the country database. At least it will help as a form of motivation, because when you are doing a job and they see the words are reflecting that, it will give you more zeal to do the work. (KII Participant 4)

#### Lack of Support and Capacity Building for Traditional Medicine

One participant raised the issue of further training for traditional healers and for greater collaboration between traditional and government mental health services. They reported a need to fund the Traditional Healer’s Union:

This is one of the big challenges we are facing as I speak to you: The Traditional Healers’ Union is not getting any funds from any international partner in order to push our mental activities in Sierra Leone. And the area of resource is a big problem as I speak to you, we have gotten a lot of problems because like for us now we have just established an allopathic clinic right there at our National Secretariat at Calaba Town. Very soon we will be completing one at Waterloo Rural which is now almost getting to an end. We need more support also to capacitate our members especially like me … because I have to move from district A to district B to know exactly the problems of our people that deal with mental health. (KII Participant 13)

We need total cooperation for our traditional mental health doctors in this psychiatry hospital so that when they realize this person has been diagnosed and doesn’t need any conventional doctor, automatically the traditional healer that deals with mental health will have to take over. We don’t want any bias, to say no this person should not go. Let’s come together; divided we fall and united we stand. (KII Participant 13)

## Facilitators

A number of facilitators in delivering nurse-led mental health services were identified. These are summarised in Table 2.

### Motivation

Motivation is a key facilitator for nurses to deliver mental health services adequately. Mental health nurses can be motivated to improve their skills and participate in continuous professional development. Mental health nurses can also be motivated through policies that support the efficient mental healthcare services. This has been with the support of international non-governmental organisations (NGOs) in collaboration with the MOHS.

…like I am opportune to have Kings (Kings Sierra Leone Partnership) with me and they are helping me, and the hospital that I am working in, they are also helping me, they are trying their bit. We have JSI (John Snow Inc.) that is also helping us with the work. I think those are the people that are helping. We have BBB, Building Back Better. Sometimes we conduct workshops, sometimes we conduct other things and Kings normally they send in researchers to come and see what we are doing; they were in last month. We also have CAPS (Community Association for Psychosocial Services) International, they came to see what we are doing and we have visitors from BBB, they also went to our units to see what we are doing, so these are some of the people that have been going to see what we have been doing. If we have workshops, we have the awareness raising … we normally do that every three months for our doctors and the matron and other people. (KII Participant 3)

### Knowledge Sharing

It was highlighted that knowledge sharing could impact on the skills of nurses. One to one knowledge sharing skills was suggested as a method to improve the skills of mental health nurses in the workforce. When one nurse is trained, the training can be passed on to other nurses. One participant added:

That is another challenging problem, like what I have done in the district on this issue, I have talked with the matron and they have given me one or two nurses, so whatever skill that I have learnt I would find a time to teach them. And again, whatever workshop I attend, I will make sure that I find time to teach them or share the knowledge with them so even if am not around like presently that I am here now, I have people in my office, so that is the first step. Then if I am in a board meeting wherein all the other experts are, they will still want to tell that they need to train more mental health nurses or practitioners, because one person cannot definitely work in a whole district. Even working in a community of about hundred people can be challenging. (KII Participant 5)

We are doing service for people that will work hand in hand with us. Like we have already trained some link nurses for them to know the important of these things so that we can have easy ways to do things. (KII Participant 1)

I remember … we have a workshop to review our tools and one of the mental health nurses was the one doing the presentation. I think it was very impressive hearing that young man talking and taking us through the mental health jargons and what is involved in mental health. I think that just shows that other people are learning… (KII Participant 11)

### Passion

Nurses continue to provide services despite the challenges they experience in providing mental health services. This is as a result of the passion for the job. As such, passion is a driving force and a key facilitator to nurse-led mental health service in Sierra Leone. One participant stated:

Just that I have passion for the job and the patients that I am treating; if not I should have given up from this mental health work long since. (FGD Participant 6)

I am improving my everyday work first of all, I like what am doing, I am passionate of what am doing, and everyday am expectant that I am going to see my patients. (KII Participant 3)

### Building Capacity

Whilst workforce challenges remain a significant problem, opportunities to increase the workforce, and modernise psychiatry in Sierra Leone, are viewed as important.

#### Increasing the Workforce

Increasing the current workforce is one way to improve mental health services in Sierra Leone. The current workforce is not large enough to cater for all mental health patients. As it is currently, the majority of mental health units have only one nurse stationed to carry out different tasks related to mental healthcare. However, it was indicated that the impact of the mental health services could be increased if more people are brought on board. One of the participants stated:

If I were the one implementing this service, I should have brought a lot of people on board, that is the first step. Because the districts are so big and so one person cannot cater for all those that have mental illness. One person cannot afford to advocate, sensitize, or treat. That you can’t do. That is the first step I should have taken, let people as many as possible come on board. (KII Participant 6)

I have seen that new recruits have come into play and there was some improvement in the physical structure at Kissy and I don’t know how far that improvement was extended to the district or the provinces. (KII Participant 10)

Am thankful because I am not working alone or I am not working in isolation in my unit as the district mental health nurse, am working with three volunteers in my unit. They are psychosocial counsellors; trained personalities and they have worked during war years … in Guinea. These people are all giving me their best. Which I can be able to carry out my work, they also assist me in doing ward rounds in the morning to see patients that really need counselling. Also, they help me with patients refusing to eat or not taking medication. With those helping hands they make our work easier. (KII Participant 7)

#### Modern Psychiatry

Introducing young people to the modern form of psychiatry which utilises new technologies could facilitate mental health services in Sierra Leone. The participants mention modern treatments like atypical antipsychotics, as well as reducing dependence on physical restraint, and access to psychological therapies and other treatments. This could also increase the workforce in mental health and raise funding. One participant explained:

To make mental health attractive just like any other medical profession, encourage the young ones by mentoring and then you expose them to modern psychiatry and increase funding I think that will help. (KII Participant 12)

People cannot afford the medicine that can give positive results very fast. (KII Participant 3)

## Discussion

The study aimed to investigate the barriers and facilitators to nurse-led mental health services in Sierra Leone. The findings suggest that factors such as small workforce and high workload, culture and beliefs, risks, lack of safety measures and required resources, outdated policies, poor salaries, lack of funds for medication, distance, power and influence, and stigma affect nurse-led mental health services. Factors such as poor working conditions, difficult terrain and limited financial incentives have also been reported as barrier to retention of rural healthcare workers in Sierra Leone (Wurie et al., 2016).

Factors that could facilitate nurse-led mental health services include: motivation, increasing the workforce, knowledge sharing, passion and modern psychiatry. This study contributes to the body of knowledge on mental healthcare in developing countries in understanding the impact of the challenges of mental healthcare services on effective healthcare delivery. Moreover, this is the first time such a study has been conducted within the Sierra Leonean context.

Service delivery within the MHU is widely understood to be affected by a number of challenges including human resource constraints and inadequate medication supply chains. The analysis provides insight into why the above factors are difficult to address and improve, and what the potential opportunities are within the system to develop the new mental health services further.

Small workforce and high workloads have always been a challenge in the Sierra Leone workforce and there are widespread issues in the recruitment and retention of nurses generally, especially outside of Freetown (Wurie et al., 2016). In the past there was only one psychiatrist (Alemu et al., 2012), although there are currently two full time Sierra Leonean psychiatrists in the country (Harris et al., 2019). In order to address this challenge and facilitate effective mental healthcare services, it is important to increase the workforce. This could be through training and education. However, the findings suggest that poor salaries deter professionals from joining the workforce. Furthermore, staff end up using their own salary to fund aspects of their work. This calls for other measures to increase the salaries of mental health nurses.

With follow up cases, community awareness raising, management activities, advocacy for their service, travel time on untarmacked roads, and potential personal issues such as illness, nurses are feeling the pressure of the workload. The training and allocation of lay health workers or healthcare assistants to assist the mental health nurses might help. Two of the participants are greatly helped by volunteers allocated to their clinics. Doctors and CHOs have some training in mental health, although this is limited. If this could be improved, or supervision structures by the psychiatrists improved, this might also support the nurses. Greater integration with the district hospitals in which the nurses work with better pooling of resources might strengthen the clinics. This might help the nurses with high caseloads, and allow those with lower caseloads to provide more community sensitisation/outreach.

The nurses in Sierra Leone are at risk through lone working and lack basic safety measures to support patients who are at risk of hurting themselves or others. Exposure of staff to high levels of risk can affect mental healthcare services though staff stress, burnout and illness, plus performance errors (Briner & Manser 2013). This is in contrast with other studies where security measures are usually put in place to support the physical safety of nurses, for example, ensuring that doors are locked and patients are closely monitored (Sierra Leone Ministry of Health & Sanitation, 2016). While nurses can adopt these measures, it is ideal to have additional support staff trained in prevention and management of violence and aggression to aid mental health nurses when dealing with challenging patients, especially when there is only one nurse assigned to one mental health unit. Undertaking home visits alone also puts the nurses at greater risk. Security measures should be put in place to support nurses in different districts in addition to increasing the workforce. Furthermore, improving medication supplies would improve the safety within mental health services with reduced rates of violence (Swanson et al., 2008).

Nurses believe that medication, in addition to therapy, could improve patients’ mental health but is often unavailable due to lack of funds. This is supported by a quantitative evaluation of the MHU carried out in the same period, which found that 25% of prescriptions were for medications that were unavailable to the patient (Hopwood et al., 2021 Apr). Reports of fake medications are backed up by the WHO, and accounting firm PricewaterhouseCooper (PwC) which has stated that up to 70% of medications in developing countries can be fake (British Broadcasting Corporation (BBC) 2020; PWC 2017). To prevent deteriorating conditions of patients, the government should either make mental health medications affordable or provide access to these patients free of charge.

To support mental health services in Sierra Leone, new mental health policies and legislation should be put in place. These policies should redefine new measures to improve mental healthcare services in Sierra Leone. Distance is usually a problem, as patients are often located in different areas far from district mental centres. Resources such as vehicles and secure ambulances should be provided to district mental health centres to facilitate the transportation of patients from different locations to the district mental health centre. New mental health legislation will need to offer greater protection for patients whilst also considering the limited workforce. This will be vital if it is to be successfully implemented. New legislation, which includes directions for treatment and rehabilitation rather than criminalising mental illness, may help to drive mental health services reform, reducing potentially abusive practices thus improving confidence in the profession, and promote the development of community services: South Africa's 2002 Mental Health Care Act states that "persons providing care, treatment and rehabilitation services must provide such services in a manner that facilitates community care of mental health care users." (Government Gazatte, 2002; UK Government, 2009).

Mental health services are often given low priority especially in regions such as West Africa (Prince et al., 2007). The findings suggest that mental health services need more attention and resources to support their effective functioning. To address the low priority given to mental health services by governments, effective advocacy is needed, including international agencies such as the WHO, along with incorporating mental health as a public health agenda, and the use of champions with financial and administrative authority (Thornicroft et al., 2010). While one or two nurses felt well supported in their units by NGOs, these NGO programs have now ended, demonstrating the need for local improvement and drivers of change.

To support nurse-led mental health services in Sierra Leone, knowledge sharing is important. Knowledge sharing mechanisms can improve the learning process by facilitating one-to-one sessions with trained and untrained nurses, thereby building capacity. Mentorship programs should also be provided to nurses to ensure adequate support and supervision. Clinicians should be involved in service design and planning to improve morale and leadership. Investment in career-long continuing training programmes and out of area professional meetings is needed (Thornicroft et al., 2010). Experiences from the roll-out of task-sharing services within primary care have identified that the success of these mental health services depends upon having sufficient staff support, compensation and supervision (Mendenhall et al., 2014). Finally, anti-stigma programmes with service user involvement and testimonies can be used to help engage communities and improve mental health literacy (Pinfold et al., 2005).

It is the view of the traditional healer that traditional medicine should be integrated into the government mental health system in terms of resources and funding. However, while the mental health nurses said they understood traditional beliefs and had professional relationships with some traditional healers, they indicated they believe the treatment provided by traditional medicine, whilst being the default for many people, is not always effective enough. They view increasing community awareness of the treatment options as the solution.

## Strengths and Limitations

The main strength of our study was the use of a qualitative method to capture the experiences of the participants and their perceptions of the barriers and facilitators to mental healthcare delivery in this novel intervention—decentralised nurse-led mental healthcare provided by trained non-specialists within secondary care services. Our preconceptions might have affected our interpretation of the findings. Three of the authors are psychiatrists with experience working in clinical or supervisory roles in Sierra Leone. The other two authors are a researcher and psychiatrist with research experience in Sierra Leone. The authors’ knowledge of the Sierra Leone mental health system is potentially both a strength and a limitation; the authors’ knowledge helped with understanding the context, but might have affected their interpretation. A further limitation was that patients’ perspectives were not included in the study. However, the perspectives of the clinical staff and managerial staff interviewed were instrumental to understanding and improving the delivery of MHU healthcare.

## Conclusions

This study investigated the barriers and facilitators of delivering decentralised mental healthcare through trained, non-specialist nurse-led clinics in Sierra Leone, using key stakeholders in the Sierra Leonean healthcare context. Based on the interviews several themes emerged such as challenging working conditions, culture, beliefs and stigma, and lack of resources, but strengths in terms of motivation, knowledge sharing, passion and building capacity. Identified challenges could be mitigated in various ways such as improving salary structures for mental health nurses in Sierra Leone, improving the availability of resources such as medications and other essential items and increasing the workforce to cater for more patients. The staff could be further supported by the allocation of trained lay workers and healthcare professionals from the district hospitals, better pooling of resources with these hospitals, strengthened systems for supervision with Sierra Leonean doctors with specific training and psychiatrists, as well as peer supervision, and improved policy and legislation coupled with investment to improve quality of care and safety. Further investment, however, must be accompanied by advocacy to improve the demand for improved services and mental health awareness among policymakers. We encourage future studies to build on and refine our investigation of this novel intervention.

## Data Availability

The datasets generated and/or analysed during the current study are not publicly available due [REASON WHY DATA ARE NOT PUBLIC] but are available from the corresponding author on reasonable request.

## Abbreviations

LMIC: Low- and middle-income countries
mhGAP: Mental Health Gap Action Plan
MHU: Mental Health Unit
MOHS: Ministry of Health and Sanitation
SLPTH: Sierra Leone Psychiatric Teaching Hospital
WHO: World Health Organisation
